# Fermentative Disorders of the Alimentary Canal of Infants

**Published:** 1900-05

**Authors:** L. G. Frazier

**Affiliations:** Port Norfolk, Va.


### FERMENTATIVE DISORDERS OF THE ALIMENTARY CANAL OF INFANTS.†

By L. G. FRAZIER, M.D.,
PORT NORFOLK, VA.

The months of July, August and September bring graver troubles among the little ones than any other season of the year. During these months there is much digestive disturbance among infants. The delicate organism is very susceptible to both internal and external agencies. We have all been duly impressed with the fact that some fermentative principle was at the bottom of almost all of the intestinal troubles among the children from six months to two years old, and that many of the intestinal troubles of larger growths and also of adults could properly be traced to some cause due to some fermentative disorder of the alimentary tract. Under the head of diseases that are caused by fermentation in the alimen-

*"In 21 of 253 operations neither perforation nor serious hemorrhage was found. It is to be doubted whether so large a number of traced penetrating bullets could fail of more serious injury, and there is a presumption of faulty diagnosis in some of them. However, they all recovered; so the operation did no harm."—*Grant.*

† Read before the Tri-State Medical Societies of Virginia and the Carolinas in Charleston, February 20, 1900.

tary canal cholera infantum is one of the most common. In the majority of the cases the trouble lies in the primary digestion or assimilation of the food.

The exciting cause is no doubt due to an acid fermentation of the food, causing excessive development of micro-organisms, either before, during or after digestion. Dentition has been considered by many writers to be a prime factor in producing these alimentary disturbances, by deranging the digestive system. From my own experience, I believe it to be a secondary condition. It is true that dentition influences and aggravates the existing bowel troubles, and in some instances may give rise to these troubles.

Some of the etiological factors we find more or less associated with fermentative disorders of the alimentary canal of infants are:

1st. Bad sanitary surroundings, impure water, uncleanliness, filth of all kinds; such as decaying organic matter, filthy stables, and stagnant water, cesspools, over-crowded tenement houses, with obstructed sewers and neglected ventilation. Among the poor who occupy crowded apartments, where the streets are extremely narrow and filthy, and the water is scanty, ofttimes contaminated with existing putrescent organic matter, we find much of this trouble.

2d. The extreme heat of summer. As long as the atmospheric temperature is mild, the intestinal disorders are moderate, but with a rise of temperature during the hot months we often find that children who a few hours ago were well and healthy, will have all the positive symptoms of cholera infantum or ileocolitis.

3d. Improper diet and feeding are always a favorable cause of these disorders, or fermentative disorders. Not one mother in a hundred understands the art of properly feeding babies. They let them take all they will from the breast or bottle into their stomachs, consequently they take in far more than they can digest. During the hot months a child is more than apt to be fretful, peevish and cross, especially if teething, and to quiet it the average mother will give it the breast or bottle just as often as it will take it. The little one will often take it for no other purpose than to rub and press the congested gums against the nipple. Yet by this means the child takes an over amount of milk into its stomach—more than can possibly be digested. Consequently, the milk undergoes fermentation and putrefactive changes, producing autoinfection. On the other hand a great many children suffer from insufficient feeding, the quality of the food is too poor and the quantity not sufficient.

4th. Dentition has been spoken of, and I will not consume space just here for a repetition. Pure water, fresh air, and plenty of sunlight (and I mean by sunlight outdoor exercise), and most important of all, proper feeding, are prime factors in treating babies who are continually sick and disturbed by some fermentative disorders of the alimentary canal. Again, children often suffer from thirst. The little ones get thirsty just as we do. I do not believe in withholding water from them. Give them plenty of water, and water that has been boiled or purified. Bottle-fed babies should always be given milk that has been boiled with a little water and sugar added. If there is the slightest symptom of indigestion, or fermentation, add a few grains of pure scale pepsin. As to the therapeutic treatment of these so-called alimentary disorders, due to fermentation of food in the canal, first evacuate the stomach and bowels of the matter that has undergone fermentation and putrefactive changes as quickly as possible, and fortunately for the little ones, nature has often done this part of the treatment in the way of emesis before the physician sees the patient. The following drugs, given in proper doses answer my purpose: Bismuth subnit., mist. cretae, syr. rhei aromat. In stubborn cases nitrate of silver; also tannic acid is used, camph. tinct. opii to relieve pain. I have gotten some good results from the use of papine to relieve pain.

Borolyptol and Listerine act splendidly as antiseptics in those cases. External applications, such as warm poultices, oftentimes relieve the pain without the aid of opiates, and should be used in preference. Many other things I might mention in connection with the treatment, such as rectal injections, sponging, and inunctions, but not wishing to bore you with a lengthy paper, I leave the subject with you, trusting I have spoken some word worthy of your thought.

